# Using Iterative Correction to Improve the Accuracy of the Blind-Hole Welding Residual Stress Test

**DOI:** 10.3390/s24196243

**Published:** 2024-09-26

**Authors:** Jiangning Pei, Shengbao Wang, Songlin Qin, Zhijia Yu, Jianzhong Gao, Jichen Xiao, Zhonglong Li

**Affiliations:** 1 School of Architecture and Civil Engineering, Harbin University of Science and Technology, Harbin 150080, China; pjn18366029511@163.com (J.P.);; 2 Longjian Science and Industry (Heilongjiang) Company Limited, Harbin 150009, China; 3 China Railway Shanhaiguan Bridge Group Co., Ltd., Qinhuangdao 066299, China; 4 School of Transportation Science and Engineering, Harbin Institute of Technology, Harbin 150090, China

**Keywords:** blind-hole drilling method, residual stress, stress release coefficient, iterative correction, secondary development

## Abstract

An iterative correction method for the stress release coefficient, leveraging numerical simulation, has been innovatively developed to address the significant error issues associated with the blind-hole method in high-stress residual stress testing of steel structures. This method effectively reduces measurement error in high residual stress regions through a discriminant iteration process. The finite element analysis technology was employed to accurately simulate the blind-hole method’s test process, and additionally, Python was utilized for customizing the secondary development of ABAQUS software, thereby automating and optimizing the method. When compared with simulation and experimental data from the welding process, the efficiency, accuracy, and reliability of the correction method have been verified. The proposed method eliminates the need for tedious calibration experiments inherent in traditional methods, significantly enhancing the test’s automation level and convergence speed, and ensuring measurement accuracy, which provides an innovative solution for the accurate evaluation of residual stress in steel structures.

## 1. Introduction

With the rapid development of modern industry, steel structures are widely used in various engineering structures such as bridges [1] and ships. Within these structures, the presence of residual stress significantly impacts the performance and safety. Particularly in areas subjected to high stress, such as in the vicinity of welded joints, the accurate assessment of residual stress is essential for ensuring structural reliability. The hole-drilling method, as a widely utilized technology for measuring residual stress, offers high measurement precision in certain scenarios. However, it exhibits certain limitations and inaccuracies when applied to high-stress areas [2].

Since J Mathar first introduced it in 1934 [3], the hole-drilling method has evolved into a widely recognized and highly accurate test method for residual stress measurement through technological innovation. In 1981, the American ASTM Association established this method as a standard for residual stress detection [4]. However, the method’s calculation foundation, which relies on elastic theory, restricts its application in high-stress areas, typically not exceeding half the yield strength of the material [5]. In high-stress regions, such as in the vicinity of the weld, the hole-drilling method’s test results are prone to various errors, including the eccentricity of the rotating hole, the lateral effect of the strain gauge, and the deviation of the bonding angle. These errors typically range from 5% to 15%. Although these errors can be mitigated by standardizing operations, increasing experimental counts, and enhancing instruments, the error due to plastic deformation at the hole edge from stress concentration is challenging to avoid and significantly impacts test outcomes. To address this issue, the academic community has introduced various correction methods. Zhao, Q. et al. [6] proposed a simplified formula for calculating the strain release factor, which, when combined with the hole-drilling method, allows for adjustments in factors A and B to observe how calculated residual tensile stress varies with the reduction of these values. Ajovalasit et al. [7,8,9,10] performed several experiments on both uniform and non-uniform stress fields using drilling techniques. The incorporation of the finite element method (FEM) in the measurement field has notably enhanced accuracy, particularly in non-uniform stress fields. Halabuk, D. et al. [11] developed a comprehensive program to correct plastic effects using neural networks, testing over a million stress states and demonstrating the capability to correct any combination of uniform residual stresses up to the material’s yield stress.

While these methods enhance measurement precision to some degree, they are often associated with issues such as laborious experimental procedures, expensive costs, intricate correction protocols, sluggish operation, and a propensity for inducing secondary errors. More importantly, conventional correction approaches frequently overlook the interplay among various factors, leading to suboptimal outcomes and diminished precision.

Building upon prior research, this study introduces an iterative correction method for the stress release factor grounded in numerical simulation, designed to surpass the constraints inherent in current methodologies and to enhance the efficiency, precision, and reliability of the corrective measures. The efficacy of this method is substantiated through experimental validation, thereby offering a novel approach to the precise assessment of residual stress within steel structures.

## 2. Principle of Blind-Hole Method to Test Residual Stress

**Figure 1:**
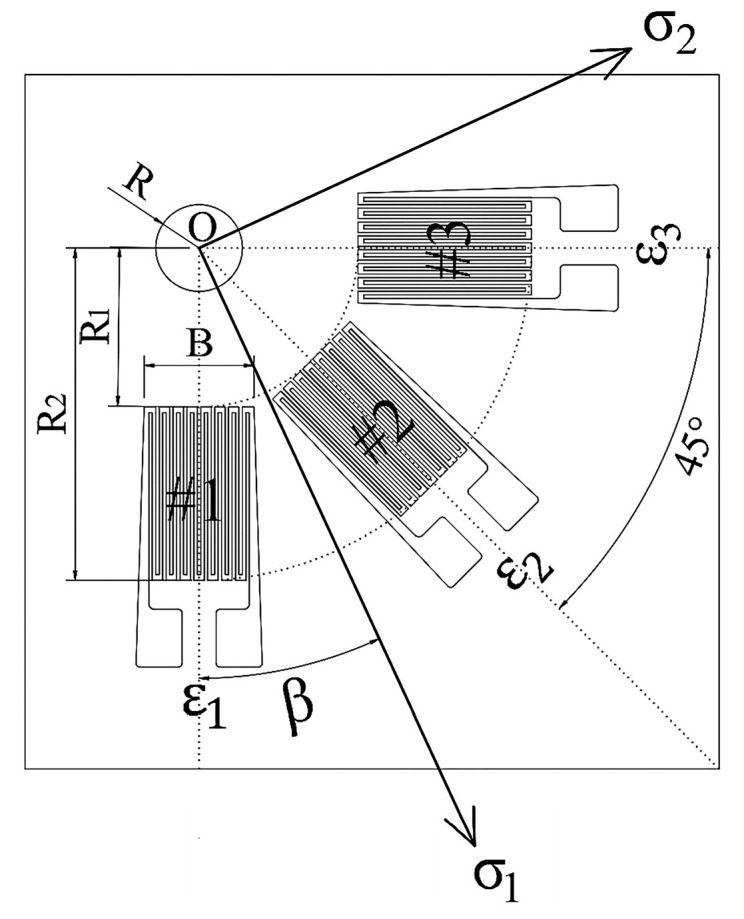
The layout of strain rosette.

The hole-drilling method is a precise technique for measuring stress, which releases local residual stress by drilling small holes into the material’s surface. During this process, a strain gauge rosette, serving as a critical measuring instrument, is meticulously applied to a predetermined point to record the strain variations that occur throughout the stress release. Figure 1 illustrates the layout of the strain gauge rosette. Once the rosette application is complete, a hole is drilled with a center at o, having a radius of r and a depth of h. After the drilling is completed, the strain values of 1#, 2#, and 3# sensitive grids measured by the strain gauge are $\varepsilon_{1}$, $\varepsilon_{2}$, and $\varepsilon_{3}$. Considering the isotropic nature of the test material and the assumption that the material behaves elastically both before and after drilling, a mechanical model is constructed based on G. Kirsch’s classical theory [12]. Consequently, the formula for calculating the residual principal stresses is established.

(1) $$\left\{\begin{array}{c} \varepsilon_{1}=A\left(\sigma_{1}+\sigma_{2}\right)+B\left(\sigma_{1}-\sigma_{2}\right)\cos2\beta \;\;\;\;\;\;\;\;\;\;\;\;\;\;\\ \varepsilon_{2}=A\left(\sigma_{1}+\sigma_{2}\right)+B\left(\sigma_{1}-\sigma_{2}\right)\cos2\left(\beta +45^{{}^{\circ}}\right)\\ \varepsilon_{3}=A\left(\sigma_{1}+\sigma_{2}\right)+B\left(\sigma_{1}-\sigma_{2}\right)\cos2\left(\beta +90^{{}^{\circ}}\right) \end{array}\right.$$

$\varepsilon_{1}$, $\varepsilon_{2}$, $\varepsilon_{3}$ —the release strain of the strain rosette 1#, 2#, 3# sensitive grid.

A, B —the stress release coefficient of strain rosette.

$\sigma_{1}$, $\sigma_{2}$—the maximum residual principal stress and minimum residual principal stress of measuring point.

where $\beta =\frac{1}{2}\tan^{-1}\left(\frac{\varepsilon_{1}+\varepsilon_{3}-{2\varepsilon }_{2}}{\varepsilon_{3}-\varepsilon_{1}}\right)$ is the direction angle of maximum residual principal stress.

Write Formula (1) in the form of $\sigma_{1}$ and $\sigma_{2}$:

(2) $$\sigma_{1,2}=\frac{\left(\varepsilon_{1}+\varepsilon_{3}\right)}{4A}\mp \frac{1}{4B}\sqrt{{\left(\varepsilon_{1}-\varepsilon_{3}\right)}^{2}+{\left(\varepsilon_{1}+\varepsilon_{3}-2\varepsilon_{2}\right)}^{2}}$$

The stress release coefficients A and B significantly influence the accuracy of the residual stress test results. Typically, these coefficients are derived from calibration experiments, with the calibration formulas provided as shown in Equations (3) and (4).

When β=0 (multiaxial stretching)

(3) $$\left\{\begin{array}{c} A=\frac{\left(\varepsilon_{1}+\varepsilon_{3}\right)}{2\left(\sigma_{1}+\sigma_{2}\right)}\;\;\;\;\;\;\;\;\;\;\;\;\;\;\;\;\;\;\;\;\;\;\;\\ B=\frac{\sqrt{{\left(\varepsilon_{1}-\varepsilon_{3}\right)}^{2}+{\left(\varepsilon_{1}+\varepsilon_{3}-2\varepsilon_{2}\right)}^{2}}}{2\left(\sigma_{2}-\sigma_{1}\right)} \end{array}\right.$$

When β=0, $\sigma_{2}=0$ (uniaxial stretching)

(4) $$\left\{\begin{array}{c} A=\frac{\left(\varepsilon_{1}+\varepsilon_{3}\right)}{2\sigma }\\ B=\frac{\left(\varepsilon_{1}-\varepsilon_{3}\right)}{2\sigma } \end{array}\right.$$

Because the material testing machine in the calibration experiment can only do uniaxial tension, the calibration experiment can only obtain the stress release coefficients A and B in the case of one principal stress, and the actual stress state on the surface of the measured point is the maximum principal stress and the minimum principal stress at the same time. Therefore, Formula (4) obviously ignores the influence of stress ratio Ω and direction angle β on stress release coefficients A and B.

## 3. Finite Element Calibration of Stress Relief Coefficient

In recent years, the finite element method has become a dominant approach for calibrating stress release coefficients. T Schajer [13], Aoh [14], Švantne [15], Zhang [16], and other scholars have employed the finite element method to calibrate the stress release coefficients A and B of the blind-hole method, achieving favorable outcomes. Therefore, ABAQUS finite element analysis software is utilized in this study to simulate the process of residual stress testing via the blind-hole method in order to determine the corresponding stress release coefficients A and B.

### 3.1. The Basic Principle of Finite Element Simulation Blind-Hole Method

In this study, the finite element simulation is the key technology for achieving precise replication of the blind-hole method test process. In the simulation process, ensuring the model accurately reflects the actual situation in terms of material properties, drilling technology, and strain measurement principle is crucial. Furthermore, the stress distribution of the model should closely correspond to the residual stress distribution on the actual specimen’s surface to ensure the accuracy and reliability of the simulation results. This process not only replicates the physical behavior of the material but also offers a novel perspective for elucidating the stress release mechanism.

#### 3.1.1. Assignment of Material Parameters

The measured material properties of the test piece (Q345qE steel) are given to the analysis model by finite element analysis software, and the material properties of Q345qE steel are shown in Table 1.

#### 3.1.2. Equivalent Application of Residual Stress

**Figure 2:**
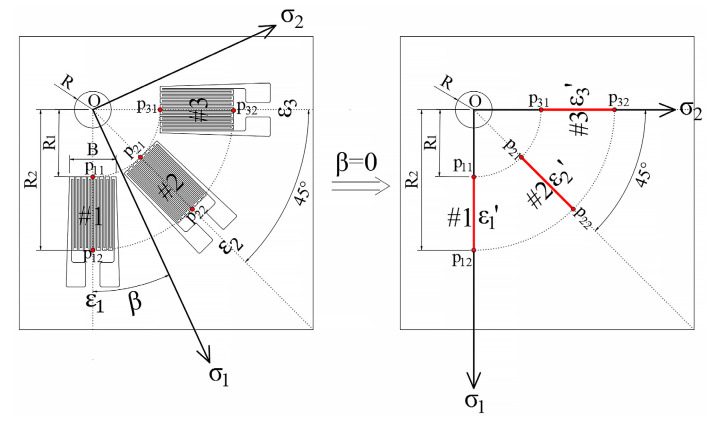
The equivalent of stress distribution state.

Incorporating the measured residual stress at the measuring point into the analysis model as the maximum and minimum principal stresses, then set the direction angle of the maximum principal stress in the model to zero before applying the principal stress, which is equivalent to aligning the 1# sensitive grid of the strain rosette along the direction of the maximum principal stress for attachment, as depicted in Figure 2.

#### 3.1.3. Simulation of Drilling Methods

**Figure 3:**
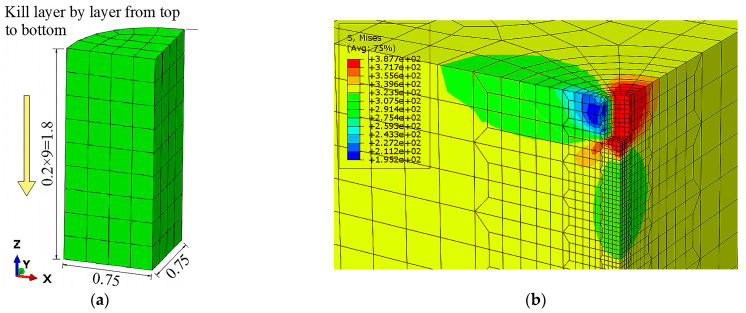
The drilling simulation process and results: (**a**) the drilling process; (**b**) the drilling results.

The killing elements method is used to simulate the actual drilling process. There are 9 layers of killing elements in total, and each layer is 0.2 mm, so the drilling depth is 1.8 mm, as shown in Figure 3.

#### 3.1.4. Extraction of Strain Relief from Strain Rosette

**Figure 4:**
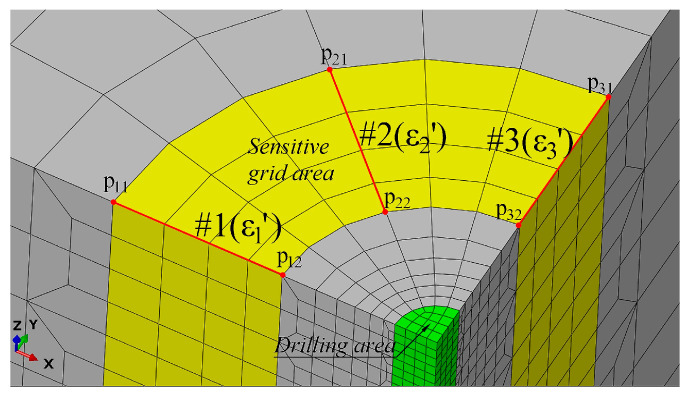
The equivalent model of strain rosette.

Use the relative distance of the P11-P12, P21-P22, and P31-P33 nodes (the actual length of the sensitive grid when working) and the orientation (the direction and position of the sensitive grid after the strain rosette is pasted) to replace the entire strain rosette in the actual test, as shown in Figure 4.

The principle of the whole process is that the relative distances of the three pairs of nodes P11-P12, P21-P22, and P31-P32 become $l_{1}^{\prime }$, $l_{2}^{\prime }$, and $l_{3}^{\prime }$ after the model is subjected to the principal stresses $\sigma_{1}$ and $\sigma_{2}$. After the stress is released, the relative distances of the three pairs of nodes become $l_{1}^{\prime \prime }$, $l_{2}^{\prime \prime }$, and $l_{3}^{\prime \prime }$. When there is no stress, the relative distances of the three pairs of nodes are $l_{1}$, $l_{2}$, and $l_{3}$ (that is, the initial length of the sensitive grid). Therefore, the release strains extracted from the analysis model after drilling are $\varepsilon_{1}^{\prime }$, $\varepsilon_{2}^{\prime }$, and $\varepsilon_{3}^{\prime }$, as shown in Equation (5). Finally, the extracted released strains are brought into Formula (3) to obtain the values of stress release coefficients A and B.

(5) $$\left\{\begin{array}{c} \varepsilon_{1}^{\prime }=\frac{l_{1}^{\prime \prime }-l_{1}}{R_{2}-R_{1}}-\frac{l_{1}^{\prime }-l_{1}}{R_{2}-R_{1}}=\frac{l_{1}^{\prime \prime }-l_{1}^{\prime }}{l_{1}}=\frac{\Delta_{1}^{\prime }}{l_{1}}\\ \varepsilon_{2}^{\prime }=\frac{l_{2}^{\prime \prime }-l_{2}}{R_{2}-R_{1}}-\frac{l_{2}^{\prime }-l_{2}}{R_{2}-R_{1}}=\frac{l_{2}^{\prime \prime }-l_{2}^{\prime }}{l_{2}}=\frac{\Delta_{2}^{\prime }}{l_{2}}\\ \varepsilon_{3}^{\prime }=\frac{l_{3}^{\prime \prime }-l_{3}}{R_{2}-R_{1}}-\frac{l_{3}^{\prime }-l_{3}}{R_{2}-R_{1}}=\frac{l_{3}^{\prime \prime }-l_{3}^{\prime }}{l_{3}}=\frac{\Delta_{3}^{\prime }}{l_{3}} \end{array}\right.$$

### 3.2. Finite Element Analysis Model Establishment

**Figure 5:**
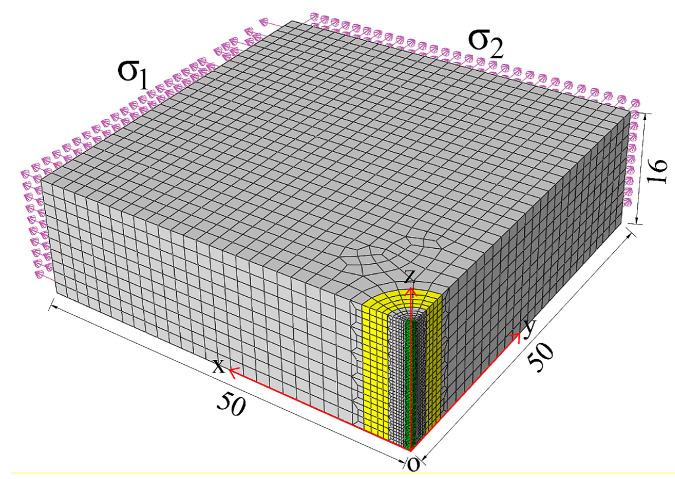
The analytical model of blind-hole method.

Since the force and boundary conditions near the drilling area of the specimen are fully symmetric in the actual drilling procedure, to enhance calculation speed and accuracy, 1/4 of the drilling area is selected as the analysis model, and the mesh of the drilling area has been refined. The dimensions of the actual analysis model are 50 mm × 50 mm × 16 mm, with symmetry constraints applied on the symmetry planes X=0, Y=0, and utilizes the eight-node linear hexahedron element (C3D8R) for meshing. The element division is illustrated in Figure 5.

### 3.3. Calibration Results of Stress Relief Factors

A small stress state ($\sigma_{1}=100\;M$Pa, $\sigma_{2}=20\;M$Pa) is applied to the analysis model, and after the model is subjected to tensile and drilling analysis according to the above method, the released strains $\varepsilon_{1}^{\prime }$, $\varepsilon_{2}^{\prime }$, and $\varepsilon_{3}^{\prime }$ of the rosettes in the analysis model are extracted and brought into Formula (3). The strain relief strain values of the blind-hole method under the low stress state are calculated as ${A=-1.29415\times 10}^{-13}\;{Pa}^{-1}$, ${B=-2.76379\times 10}^{-13}\;{Pa}^{-1}$. In addition, the strain rosette size used in the residual stress test by the blind-hole method is shown in Table 2.

## 4. Iterative Correction Method for Residual Stress

The residual stress iterative correction method proposed in this study is a systematic numerical optimization process that aims to accurately estimate the true values of stress release coefficients A and B through the measured release strain data $\varepsilon_{1}$, $\varepsilon_{2}$,$\;\varepsilon_{3}$. This process first sets a set of initial stress release coefficients and then calculates the predicted principal stress value based on these coefficients. Subsequently, these predicted values were corrected by a simulated blind-hole test to approximate the true stress state. This iterative process is repeated until the predicted stress release coefficient converges to a stable value, ensuring that the final principal stress estimation is consistent with the actual measured value.

### 4.1. The Basic Principle of Iterative Correction

**Figure 6:**
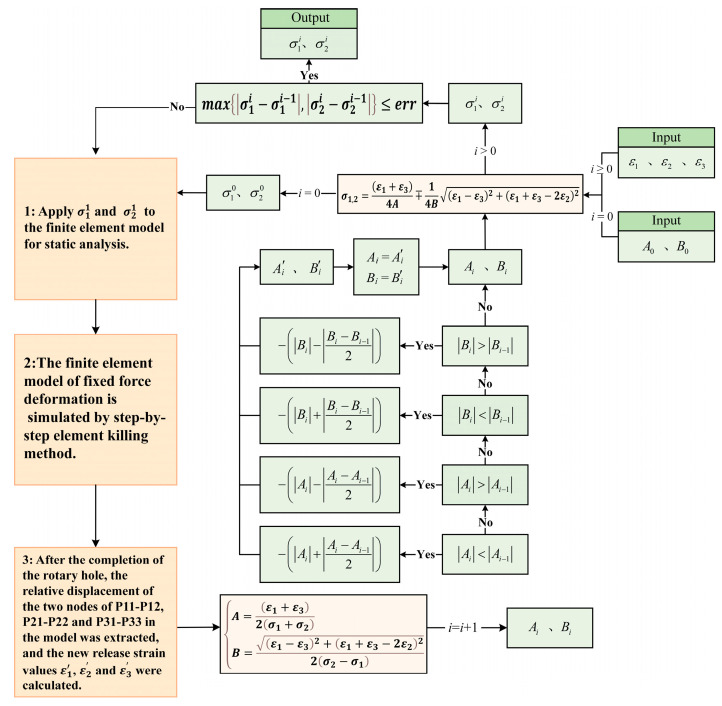
The process of iterative correction of residual stress.

The process of this iterative correction is as follows. First, an appropriate initial stress release coefficient $A_{0}$ and $B_{0}$ must be provided. Then, the actual release strains $\varepsilon_{1}$, $\varepsilon_{2}$, and $\varepsilon_{3}$ for each measuring point are obtained through the blind-hole test experiment. The initial principal stresses $\sigma_{1}^{0}$ and $\sigma_{2}^{0}$ for the corresponding measuring point are then calculated. There is a certain error between the initial principal stress calculated for the measuring point in the high-stress area and the actual principal stress. It is important to note that if the initial stress release coefficient is too small, the calculated maximum principal stress may exceed the ultimate tensile strength of the material, which is clearly unrealistic (the high residual strain zone will not be broken). Therefore, when giving the initial stress release coefficients $A_{0}$ and $B_{0}$, a limiting condition must be set: the calculated maximum principal stress must be less than the ultimate tensile strength of the material. Due to the large error between the initial principal stress value and the actual residual principal stress at the measuring point, it is necessary to further adjust the stress release coefficient and re-calculate. Thus, after subjecting $\sigma_{1}^{0}$ and $\sigma_{2}^{0}$ to the analysis model, borehole analysis is performed on the model with stress and strain (equivalent residual stress and strain), and the strains ${\varepsilon_{1}}^{\prime }$, ${\varepsilon_{2}}^{\prime }$, and ${\varepsilon_{3}}^{\prime }$ in the model are extracted to calculate the new stress release coefficients $A_{1}$ and $B_{1}$. These new coefficients are then evaluated, and appropriate corrections are made. Finally, the modified strain release coefficients ${A_{1}}^{\prime }$ and ${B_{1}}^{\prime }$, along with the actual measured release strains $\varepsilon_{1}$, $\varepsilon_{2}$, and $\varepsilon_{3}$, are used to calculate the residual principal stresses $\sigma_{1}^{1}$ and $\sigma_{2}^{1}$ after the first iteration of correction. These are compared with the initial stresses $\sigma_{1}^{0}$ and $\sigma_{2}^{0}$. If the objective function is satisfied, the calculation stops, and the true residual principal stress value is output. Through iterative correction calculations, when $\sigma_{1}^{i}$ and $\sigma_{2}^{i}$ meet the objective function after *i* iterations, the true residual stress of the measured point is obtained. The specific calculation process is shown in Figure 6.

### 4.2. Optimization of Iterative Correction Method

When performing residual stress tests in high-stress areas, manually iterating within ABAQUS is cumbersome and time intensive, especially for samples with a large number of measuring points, where the cumulative time required is substantially higher. In the stress concentration areas, iterations exceeding ten are often necessary to ensure accuracy, thereby potentially extending the entire test cycle to several days. To enhance the efficiency of this process, this paper introduces an optimized iterative correction method developed using the Python programming language. The method encapsulates the iterative process into a modular subroutine and employs loops and conditional statements to facilitate an automated iterative process, significantly reducing human intervention and the iteration time for individual measuring points.

To further enhance the automation of iterative correction, this study integrates Python scripts capable of performing a series of subroutines. These subroutines collaborate to achieve a fully automated process, from the commencement of iteration to the attainment of predetermined accuracy, utilizing looping and conditional control mechanisms. This enhancement significantly reduces the duration of a single iteration and decreases the correction time for each measuring point to under 20 min. Furthermore, aiming to minimize user intervention, this study developed an automated data input and output mechanism, enabling the automated reading and processing of the released strain values from the measuring points by analyzing test data in the Excel spreadsheet. Upon executing the script within the ABAQUS environment, the software then automatically identifies and analyzes the relevant data in the Excel file, performs iterative corrections on each measuring point sequentially, and updates the final residual principal stress values back to the Excel spreadsheet, thereby achieving efficient and convenient data management.

**Figure 7:**
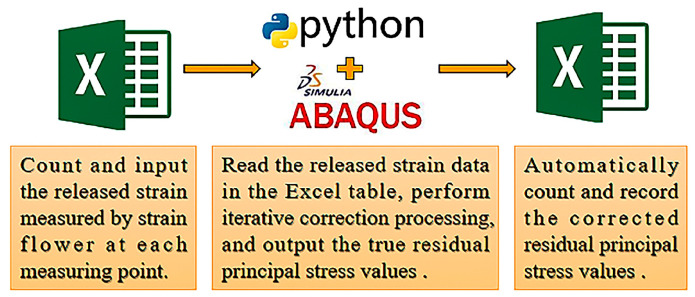
The overall process of optimizing iterative correction method.

The optimization of the iterative correction method in this study significantly reduces the test cycle for the entire specimen and reduces the workload duration from several days to just a few hours. The optimized process enhances the accuracy of corrections while also simplifying the operational process and improving overall efficiency by minimizing manual operations. The iterative correction process comprises three sequential and efficient stages: data input, automatic correction, and result recording. The optimization of this process derives advantages from methodological innovations and the collaborative work of software tools, as illustrated in Figure 7.

## 5. Verification of Residual Stress Iterative Correction Method

To thoroughly assess the effectiveness and accuracy of the iterative correction method in measuring residual stress, this study employed a comprehensive set of three methods: welding tests, numerical simulations of welding, and the blind-hole method.

### 5.1. Test Piece Welding Test

**Figure 8:**
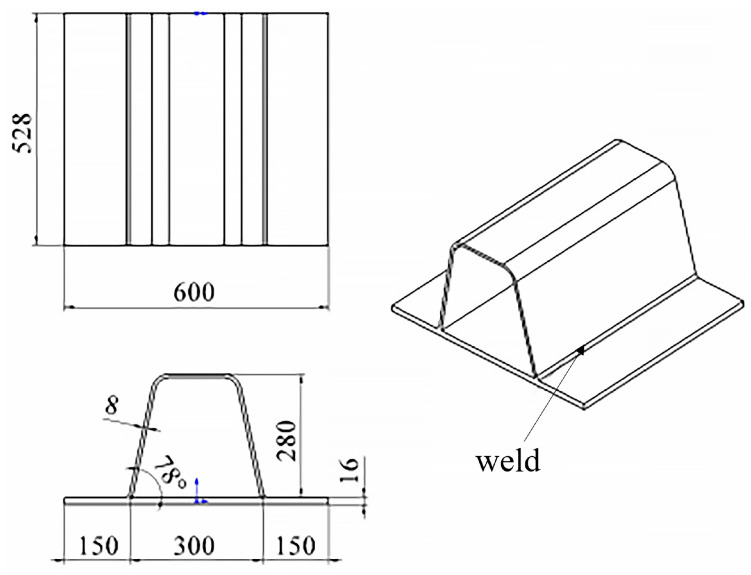
The size of welding test piece.

The setup of the welding test is crafted to mimic a high-stress environment akin to actual engineering applications for the blind-hole method test. The U-rib stiffened bridge deck serves as the research object, with dimensional details depicted in Figure 8. The welding process follows the previous research [17]. Automatic submerged arc welding technology is employed. The welding voltage is 34 V ~ 36 V, the welding current is 560 A ~ 568 A, and the welding speed is 420 mm/min, which ensures the standardization and repeatability of the welding process. This procedure not only simulates the high-stress state in practical scenarios but also supplies the necessary experimental data and conditions for subsequent numerical simulations and blind-hole tests.

**Figure 9:**
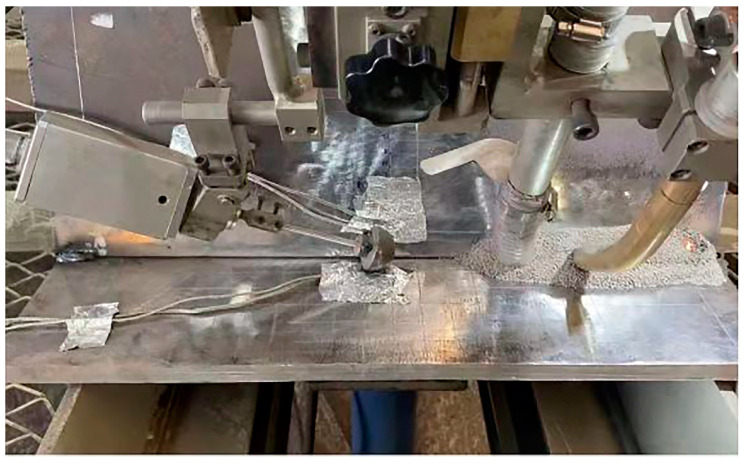
The process of fully automatic submerged arc welding.

The precisely machined specimen is positioned on the custom U-rib welding jig. To ensure the stability and repeatability of the welding process, a specialized clamping device secures the specimen to preclude any displacement or vibration. Prior to welding, all parameters have been meticulously calibrated to ensure precise control over the entire process. Figure 9 illustrates the specimen’s welding procedure.

### 5.2. Welding Numerical Simulation

**Figure 10:**
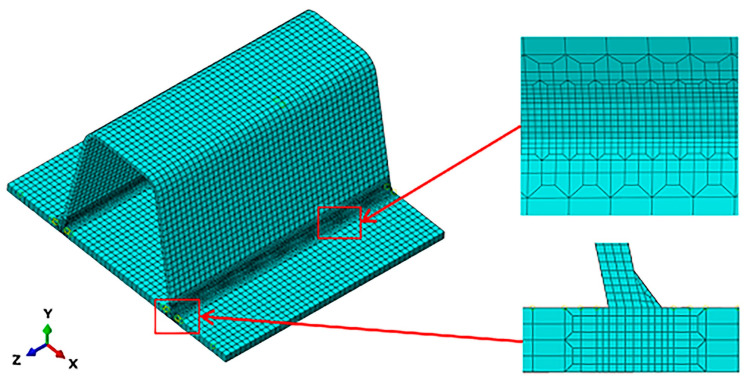
The model for welding analysis.

To directly obtain the welding residual stress field of the weldment, the study employs ABAQUS finite element analysis software for simulating the welding process of the specimen. Initially, the welding analysis model is established based on the dimensions depicted in Figure 8, and appropriate meshing is performed, as illustrated in Figure 10.

**Figure 11:**
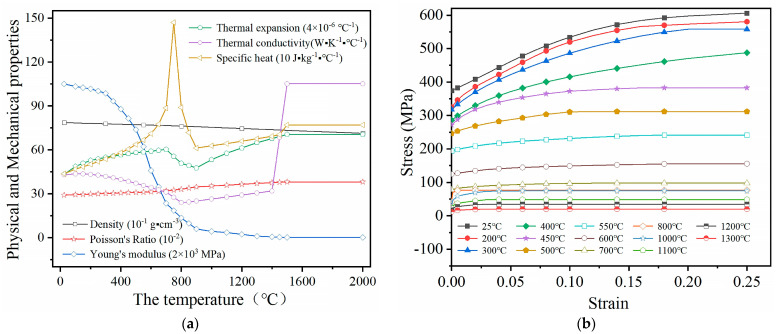
The material properties of Q345qE steel as a function of temperature: (**a**) the physical and mechanical properties; (**b**) the plastic stress strain.

**Figure 12:**
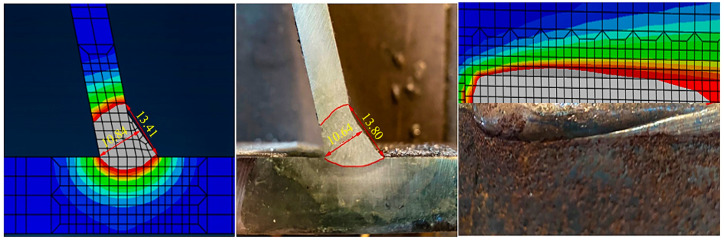
The comparison of molten pool shapes.

Owing to the absence of specific material properties of Q345qE steel at high temperatures, the thermophysical and thermo-dynamic parameters of low carbon steel, derived from the cited literature [18,19,20,21], have been organized and refined to obtain more reliable material parameters, as depicted in Figure 11. These derived material properties are assigned to the welding analysis model, followed by simulating the weld growth using the life and death element method, and the implementation of the moving heat source in welding is facilitated by a subroutine, incorporating the double ellipsoid heat source model by John Goldak [22]. Concurrently, referring to the welding process and time from the welding test, a suitable analysis step is established and the job is submitted for analysis and calculation. Finally, a comparison of the molten pool’s shape (Figure 12) verifies the consistency between the numerical simulation results and actual welding outcomes.

**Figure 13:**
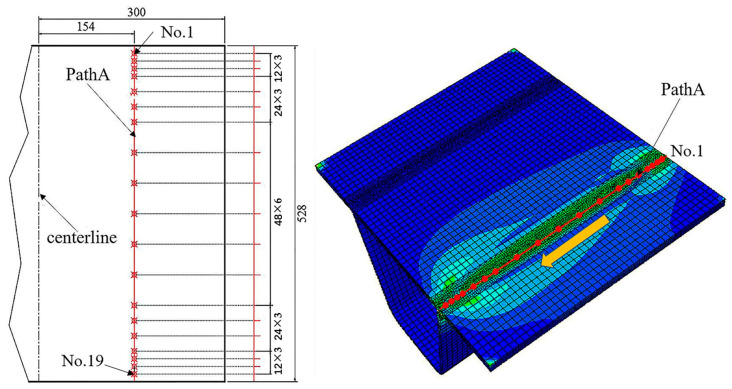
The arrangement of residual stress measuring point.

After the welding analysis is completed, the most residual principal stress values $\sigma_{1}$ and $\sigma_{2}$ of all measuring points on the PathA path of the test piece are extracted, and the layout of the measuring points is shown in Figure 13.

The residual principal stress values $\sigma_{1}$ and $\sigma_{2}$ of the measuring points cannot be directly extracted from the welding analysis results, so they need to be converted by Formula (6) [23]. The values of $\sigma_{x}$, $\sigma_{z}$, $\tau_{xz}$, and $\tau_{zx}$ were extracted from the analysis results, and then brought into Formula (6) to obtain the residual principal stress values $\sigma_{1}$ and $\sigma_{2}$ of all measuring points on the PathA path. The calculation results are shown in Table 3.

(6) $$\sigma_{1,2}=\frac{\sigma_{z}+\sigma_{x}}{2}\pm \sqrt{\frac{{\left(\sigma_{z}-\sigma_{x}\right)}^{2}}{2}+\frac{{\left(\tau_{zx}-\tau_{xz}\right)}^{2}}{2}}$$

### 5.3. Blind-Hole Method Test Residual Stress Test

**Figure 14:**
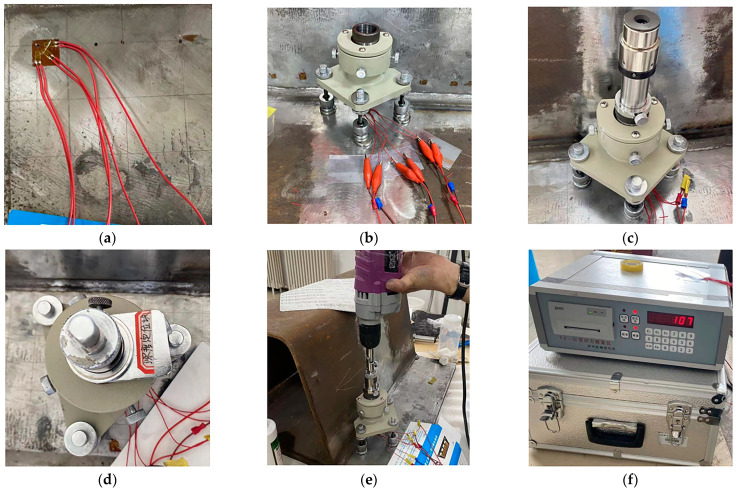
The process of testing residual stress by blind-hole method: (**a**) paste of the strain rosette; (**b**) Fixed drilling support; (**c**) align the measuring point; (**d**) control drilling depth; (**e**) drilling of measuring points; (**f**) record data.

When the test piece is welded and cooled to room temperature. Referring to GB/T 31310-2014 “Determination of Residual Stress in Metal Materials—Drilling Strain Method” [24], the blind-hole method is used to measure the release strain values $\varepsilon_{1}$, $\varepsilon_{2}$, and $\varepsilon_{3}$ of all measuring points on the PathA path. The operation process is shown in Figure 14.

The released strains $\varepsilon_{1}$, $\varepsilon_{2}$ and $\varepsilon_{3}$ measured at each measuring point are brought into Formula (2) with the calibration ${A=-1.29415\times 10}^{-13}\;{Pa}^{-1}$, ${B=-2.76379\times 10}^{-13}\;{Pa}^{-1}$ coefficients, respectively, and the uncorrected residual principal stress values $\sigma_{1}$, $\sigma_{2}$ are obtained, as shown in Table 4.

Table 4 is the test residual principal stress value of the measuring point on PathA. It can be seen from Table 4 that the minimum residual principal stress value of each measuring point is within half of the yield strength of the material, but the deviation from the minimum residual principal stress value calculated in Table 3 is large. In addition, the maximum principal stress of No. 3–18 measuring points All are larger than the maximum principal stress value calculated in Table 3, and the maximum principal stress of No. 6, 7, and 10 measuring points even exceeds the ultimate tensile strength of the material Q345qE ($f_{m}=610$ MPa). This is obviously inconsistent with the actual situation, so the residual stress value in the high-stress area calculated by the stress release coefficient under the low-stress state will be seriously large, and it is also confirmed that the blind-hole method cannot be used for the residual stress test in the high-stress area.

### 5.4. Comparative Analysis of Residual Stress before and after Correction

**Figure 15:**
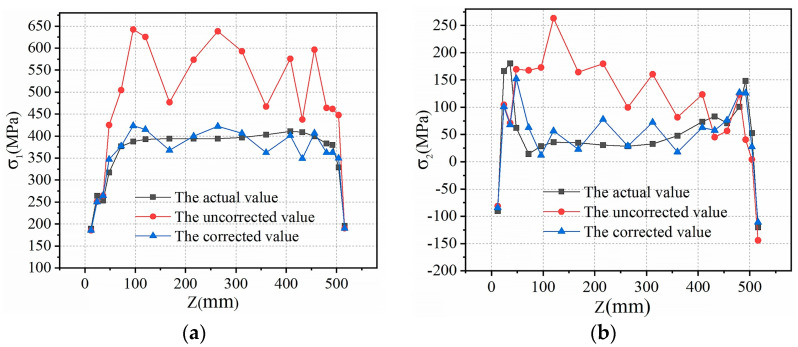
The comparison of residual principal stress values before and after correction with actual values: (**a**) the maximum residual principal stress; (**b**) the minimum residual principal stress.

Table 4 shows there is an error in calculating the residual stress value of the measuring point with the stress release coefficient under the low stress state. Therefore, the released strains $\varepsilon_{1}$, $\varepsilon_{2}$, and $\varepsilon_{3}$ of all measuring points on the PathA path can be entered into Excel, and set the allowable error value err=1, then run the iterative correction program proposed in this paper in ABAQUS for automatic correction, and finally compare the corrected results of all measuring points, the results before correction, and the results of numerical analysis (actual values), as shown in Figure 15.

Figure 15 illustrates that after all measuring points have been corrected using the residual stress iterative correction method introduced herein, the corrected residual principal stress closely aligns with the actual residual principal stress values. Nevertheless, influenced by factors including drilling inaccuracies, strain gauge adhesion, and surface finishing at measurement points during the blind-hole method test, individual measurement point corrections may deviate from actual values, although they exhibit minor fluctuations around actual values. 

**Figure 16:**
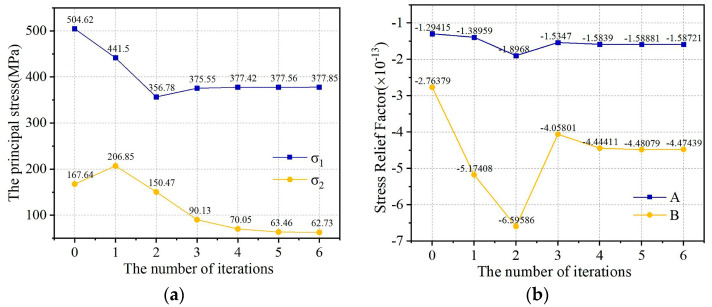
The variation in residual principal stress and stress release coefficient with iteration times: (**a**) the principal stress; (**b**) the stress release coefficient.

To explore the trends in residual principal stress and stress release coefficient as the number of iterations increases, the study focuses on measuring point 5 as the subject of investigation. It employs the iterative residual stress method for correction, recording the updated principal stress values after each iteration and the new stress release factor, thereby determining the relationship between the updated principal stress, the new stress release factor, and the iteration count, as illustrated in Figure 16.

As can be seen from Figure 16 after 6 iterations of this measuring point, the maximum residual principal stress decreased from 504.62 MPa to 377.85 MPa, and the minimum residual principal stress decreased from 167.64 MPa to 62.73 MPa. With the increase in the number of iterations, the tolerance of the two is gradually reduced until it stabilizes to within the error range. For the stress release coefficients A and B, after 6 iterations, the absolute values of the final coefficients A and B both increased and stabilized to a certain value, and the coefficient A changed more than the coefficient B.

## 6. Conclusions

This study numerically simulates the process of testing residual stress using the blind-hole method. Grounded in the fundamental principles of the blind-hole method, an iterative correction method for residual stress is proposed, utilized for correcting errors in residual stress values within high-stress regions as determined by the blind-hole method. Concurrently, the ABAQUS and Python software are employed to program and automate the correction process, thereby ensuring that the speed of iterative correction is enhanced and the results are more precise. Finally, welding tests, numerical simulations, and blind-hole method stress measurements are conducted, and comparing the residual principal stress values derived from numerical analysis with those from the blind-hole method before and after correction, the following conclusions are drawn:

In this paper, A and B are calibrated by the finite element method, and the stress release coefficient of the strain rosette under the low stress state of the residual stress experiment is obtained as ${A=-1.29415\times 10}^{-13}\;{Pa}^{-1}$, ${B=-2.76379\times 10}^{-13}\;{Pa}^{-1}$. It is found that the residual stress value in the high stress area (weld area) calculated by the calibrated stress release coefficient under the low stress state will be seriously large and even exceed the maximum tensile strength of the material.

The welding process of the U-rib stiffened plate was numerically analyzed by ABAQUS finite element analysis software, and the comparison of the molten pool profile confirmed that the numerical analysis results were in good agreement with the actual welding results.

Aiming at the problem of large residual stress error in the high-stress area tested by the blind-hole method, this paper proposes an iterative correction method for residual stress, and optimizes it, so that the speed of iterative correction is increased by about 93%. The residual stress of each measuring point is corrected, and the residual principal stress value after the corrected value is basically consistent with the real value. Finally, through the corrected residual stress, it is found that the maximum residual principal stress value at the weld exceeds the yield strength of the material.

In the process of using the residual stress iterative correction method to correct the measurement points, with the increase in the correction iterations, the error range is continuously reduced, $\sigma_{1}$, $\sigma_{2}$, and A, B each tend to a certain value, which shows that the modified iterative method is convergent.

## Figures and Tables

**Table 1:** The material properties of Q345qE steel.

ρ (g/cm^3^)	μ	E (GPa)	$f_{y}$ (MPa)	$f_{m}$ (MPa)
7.85	0.29	2.09	374	610

ρ—density.  μ—Poisson’s ratio.  E—Young’s modulus. ${\;f}_{y}$—yield strength. ${f\;}_{m}$—tensile strength.

**Table 2:** The size and specification of strain rosette.

a (mm)	$r_{1}$ (mm)	$r_{2}$ (mm)	b (mm)
0.75	2.75	5.75	1.90

**Table 3:** True residual principal stress values at measuring points on PathA path.

Measuring Point	$\sigma_{x}$ (MPa)	$\sigma_{z}$ (MPa)	$\tau_{xz}$ (MPa)	$\sigma_{1}$ (MPa)	$\sigma_{2}$ (MPa)
1	180.15	−125.54	−54.47	189.57	−89.95
2	248.60	182.34	−36.02	264.40	166.53
3	248.26	185.34	−18.15	253.13	180.47
4	317.54	62.20	6.15	317.68	62.05
5	375.95	15.02	15.63	376.63	14.34
6	387.38	29.14	11.49	387.75	28.78
7	392.33	36.02	9.37	392.58	35.78
8	394.17	34.93	6.60	394.30	34.81
9	393.92	30.61	5.69	394.01	30.52
10	394.09	28.61	6.31	394.19	28.50
11	396.58	32.90	7.82	396.75	32.73
12	402.76	48.00	10.10	403.05	47.71
13	410.18	73.94	12.59	410.65	73.47
14	408.56	83.79	14.36	409.20	83.15
15	398.45	71.85	17.21	399.35	70.94
16	378.51	104.83	35.01	382.92	100.42
17	368.04	160.06	50.61	379.70	148.40
18	312.26	69.01	65.47	328.76	52.51
19	173.59	−106.92	81.74	195.67	−120.00

**Table 4:** The test residual principal stress value of the measuring point on PathA.

Measuring Point	$\varepsilon_{1}$ (×10^−6^)	$\varepsilon_{2}$ (×10^−6^)	$\varepsilon_{3}$ [×10^−6^]	$\sigma_{1}$ [MPa]	$\sigma_{2}$ [MPa]
1	−86	0	59	185.57	−81.26
2	−85	−35	−7	251.03	104.41
3	−97	−41	10	264.96	71.17
4	−141	−107	−13	425.36	169.62
5	−180	−92	6	504.62	167.64
6	−235	−97	24	642.39	172.82
7	−215	−120	−15	625.44	263.17
8	−169	−91	3	476.93	164.42
9	−206	−88	11	573.73	179.66
10	−244	−85	53	638.29	99.64
11	−217	−99	22	592.90	160.49
12	−172	−105	30	467.11	81.52
13	−214	−71	33	575.84	123.46
14	−161	−108	36	437.76	45.18
15	−233	−70	64	596.40	56.54
16	−170	−69	18	464.16	123.10
17	−175	−103	45	461.67	40.59
18	−178	−86	61	447.86	4.18
19	−95	−31	83	190.42	−144.06

## Data Availability

The original contributions presented in the study are included in the article, further inquiries can be directed to the corresponding authors.
